# A cross-sectional study of COVID-19 impacts in culturally and linguistically diverse communities in greater Western Sydney, Australia

**DOI:** 10.1186/s12889-021-12172-y

**Published:** 2021-11-13

**Authors:** William Mude, Clement Meru, Carolyne Njue, Rebecca Fanany

**Affiliations:** 1 School of Health, Medical and Applied Sciences, Central Queensland University, Cairns Central, QLD 4870 Australia; 2 SydWest Multicultural Services, Western Sydney, Australia; 3 The Australian Centre for Public and Population Health Research (ACPPHR), University of Technology Sydney, Sydney, Australia; 4 School of Health, Medical and Applied Sciences, Central Queensland University, Melbourne, Australia

**Keywords:** Australia, CALD communities, Impacts of COVID-19, Lives domains, Migrant and refugee communities, Resilience and coping, Social services’ disruptions

## Abstract

**Background:**

This study explored the experiences of people from culturally and linguistically diverse (CALD) backgrounds in Greater Western Sydney, Australia, in selected livelihood items during COVID-19 and the perceived impacts of the pandemic on their lives.

**Methods:**

A cross-sectional survey was used to collect data between 25 August and 30 September 2020 among CALD communities in Greater Western Sydney. Information was collected on respondents’ experiences in selected livelihood items, including housing, finances, safety, accessing social services and activities, finding work, food, clothing, and relationships during COVID-19 and the pandemic’s perceived impact on their lives. Descriptive and non-parametric statistics were used to analyze the data.

**Results:**

One hundred and ninety-eight participants were included in the study, 45.5% male and 54.5% female. Many respondents reported their experience in the selected livelihood items as “worse” during COVID-19 at the time of the study. The feeling of safety was most disrupted, with 56% of respondents rating their experience as “worse”. This experience was followed by accessing social support group activities, with 50% of respondents stating their experience of using this service had worsened. The experience of accessing social services and financial situation was rated as “worse” by 41% of respondents. Experience in finding work, housing, and attending schools were all rated as “worse”. The median perceived impact of COVID-19 among respondents who rated their experience in the selected livelihood items as “worse” were statistically higher than those who rated their experience as the “same”. Respondents’ characteristics also predicted the perceived impact of COVID-19. Unemployed respondents were 3.53 (95% CI: 1.16–10.73, *p* = 0.026) times more likely to perceive the impact of COVID-19 on their lives as “high” compared to employed respondents.

**Conclusions:**

The finding demonstrates that the “high” number of respondents had the same situation as before COVID-19 and highlights the level of resilience exhibited by CALD communities in the Australian context. It also suggests that services in Australia were good during the pandemic. However, enhanced policy and initiatives designed to meet the CALD population’s needs are required, particularly in the areas most reported to have been disrupted by changes associated with COVID-19.

**Supplementary Information:**

The online version contains supplementary material available at 10.1186/s12889-021-12172-y.

## Introduction

The COVID-19 pandemic has caused considerable disruptions and uncertainties in people’s lives globally [1, 2]. It has disrupted trade and commerce worldwide, with countries closing borders, grounding air travel, and imposing strict quarantine laws [1, 3]. Like many other countries, there was a sharp change in Australia because of COVID-19, with the government closing borders, introducing lockdowns and social distancing regulations [4]. These regulations forced many businesses to close their doors and lay off employees, and unemployment rose from 5.2% pre-COVID-19 to 7.1% during COVID-19 in May 2020 [5].

In light of rising unemployment and financial hardship, Australian governments’ responses have followed many different trajectories to support disadvantaged and vulnerable individuals [6, 7]. The federal government has responded by providing the JobKeeper allowance, a wage subsidy program given to workers through their employers, but this subsidy was only available to employers and workers who met specific eligibility criteria under the scheme [8]. JobKeeper scheme was implemented in two phases – phase one was from 30 March 2020 to 27 September 2020, and phase two was from 28 September 2020 to 28 March 2021 [9].

The scheme was not available to unemployed people and casual employees who have been in their job for less than 12 months. It drew criticism for leaving out the people who needed the most help and putting casual employees at risk of sacking in favour of workers who have met the eligibility terms [10]. In addition, the critics of the JobKeeper scheme argued that the strict guidelines disadvantaged many vulnerable people, including people from culturally and linguistically diverse (CALD) backgrounds who are overrepresented in the casual workforce or are unemployed [4, 6]. In the Australian context, CALD commonly refers to people born overseas or have a parent born overseas or speak a language other than English at home [11]. In 2016, 28.6% of Australia’s population were born overseas, and 45% had at least one parent born overseas [11]. In Greater Western Sydney, 49% of residents spoke a language other than English at home [12].

The socio-economic challenges experienced by CALD communities in Australia are well-documented elsewhere and include poor living conditions, poverty and unemployment, and low income [13–16]. For example, a 2019 report found that 9.2% of people born overseas were unemployed compared to 4.7% born in Australia [17]. Moreover, CALD communities are over-represented in low-paid and mostly casual sectors, and many are employed in roles locals avoid [18]. For example, Colic-Peisker and Tilbury [18] found that CALD communities are over-represented in fields like cleaning, aged care, meat processing, taxi driving, security and building.

A major concern is that these sectors are hardest hit by COVID-19 globally, including in Australia [19, 20]. For example, many people have lost their jobs in taxi driving, hospitality, and food and beverages. In addition, in cleaning and aged care, many workers have been exposed to an increased risk of catching COVID-19. Bui et al. [21] report that 73% of workplace outbreak-associated COVID-19 cases in the United States were in the minority non-white populations. Although these are important studies that shed light on COVID-19 in CALD communities, limited research specifically examines the impacts of COVID-19 among people from CALD backgrounds in Australia. At the same time, understanding this issue in CALD communities is vital for effective and equitable policies and services.

Therefore, it is timely that we better understand the impacts of COVID-19 in CALD communities in Australia because, in times of crisis, such as the current COVID-19 pandemic, people who are disproportionately affected physically and emotionally, economically and socially are the disenfranchised [22]. For this and other reasons, disruptions caused by COVID-19 have heightened the need to better understand the extent of potentially growing inequalities experienced by CALD communities in Australia. Of great concern is the potential impact of COVID-19 in worsening inequalities in CALD communities, given the nationwide lockdown and the disruptions to education, employment, services, and families. In this sense, the pandemic is an opportunity to understand the challenges and the opportunities arising from social and economic disruptions in general and at present.

This study examined the potential exacerbation of inequalities caused by COVID-19 in key domains among CALD communities in the Greater Western Sydney region in Australia to contribute to national and international understanding of the impact of COVID-19 in socially disadvantaged and racial minority communities. Up to 38.6% of the region’s residents were born overseas compared to 27.6% of NSW’s population [23].

The current study was conducted in partnership with SydWest Multicultural Services and aimed to understand how CALD communities in Western Sydney experienced change due to the COVID-19 pandemic in their work, education, relationships, and access to services. In addition, this study aimed to identify critical areas needing targeted strategies and policies to address potentially exacerbated inequities experienced by CALD communities as a result of COVID-19 disruptions and also support policies and services that might mitigate the risk of ill health later in lives resulting from the disadvantages caused by the pandemic.

## Methods

This study employed cross-sectional surveys to collect data from adult CALD participants between 25 August and 30 September 2020. During this time, Sydney had daily reported COVID-19 cases in double units [24], and a Public Health Order was in place, with four-square metres social distancing rules enforced in public places, and the numbers of people in places of worship, hospitality venues, gyms, weddings, funerals and visitors restricted [25].

### Survey instrument development

The survey questionnaire was developed following extensive consultation with SydWest Multicultural Services and search of previous literature on the impacts of pandemic disease like influenza (See, for example, Rubin et al. [26], Rubin et al. [27], Kristiansen et al. [28] and many more). In addition, SydWest Multicultural Services reviewed the survey before implementation to ensure relevance, readability, and clarity of items in the instrument.

The survey items explored emerging issues relating to COVID-19 disruptions that included respondents’ experiences in key selected livelihood items, including housing, food, clothing, feeling of safety, accessing social services, schooling, attending language classes, finding work, accessing health care services, attending vocational training, relationships with partners, relationships with children, mental wellbeing, alcohol use, illicit substance use, and gambling. The survey asked respondents to rate their experience in these selected livelihood items as: “Same as before COVID-19” (Same) or “Worse than before COVID-19” (Worse), and “Not Applicable”. The reliability of these items was determined by conducting a principal component analysis. The KMO for the selected livelihood items was 0.80, and a Cronbach Alpha was 0.85, which show the instrument’s validity and internal consistency [29]. Another question item scored on a scale of 1–10 (with 1 being ‘not at all’ and 10 being ‘extremely high impact’) measured the perceived impacts of COVID-19 on respondents’ lives. The online questionnaire was designed in Qualtrics and administered in collaboration with SydWest Multicultural Services. A copy of the survey instrument is attached in Additional file 1.

### Sampling and recruitment

The respondents were sampled using convenience sampling [30] and recruited through CALD community leaders and community workers. Mixed-mode online and traditional media was used to distribute the survey [31]. The survey was distributed among community leaders and community workers from different CALD backgrounds through emails to disseminate in their communities. Online social media platforms and community media outlets were also used to distribute the survey. Respondents who could not complete the surveys online were provided with hard copies of the surveys to complete. Individuals were included in the study if they were at least 18 years old and self-identified as having a CALD background and lived within Greater Western Sydney. The online survey terminated if the respondents did not meet all these conditions and were automatically excluded from the study.

### Explanatory variables

Gender, age, region of origin, visa categories, residency status, years lived in Australia, number of children in a household, household size, employment status, work situation, work fraction, education level, and income.

### Outcomes

1. Descriptive characteristics of respondents and their experiences in the selected livelihood items: Housing, food, clothing, feeling of safety, accessing social services, schooling, attending language classes, finding work, accessing health care services, attending vocational training, relationships with partners, relationships with children, mental wellbeing, alcohol use, illicit substance use, and gambling.
2. The median difference in perceived impacts of COVID-19 between respondents who rated their experience in the selected items as “worse” and the “same”.
3. The perceived impact of COVID-19 when controlled for respondents’ characteristics.

### Statistical analysis

Only data from respondents who completed at least 50% of the survey items were considered as per the literature [32]. Variables with low response rates were included in the analysis because any information is better than no information as they shed some light on the examined items. Patterns of the missing data were assessed and were found to be randomly distributed, and the missing values were imputed using Multiple Imputations. Tests of normality found the data did not meet the assumptions for normality, and therefore, non-parametric statistics were used to establish statistical significance.

Descriptive statistics were used to describe respondents’ characteristics and experiences (Worse/Same) in the selected livelihood items. Mann Whitney U test was used to establish statistical significance in the median difference in perceived impact of COVID-19 between respondents who rated their experiences as “worse” or “same” in the selected livelihood items. A *p*-value of less than 0.05 was deemed significant. The effect size was calculated by dividing the z-tests with the square root of sample size (N) to quantify the difference in size between the two groups [33, 34]. According to Cohen [35] criteria, referenced in Fritz et al. [34], a small effect is 0.1, a medium effect is 0.3, and a large effect is 0.5. Exponentiated Ordinal Logistic Regression was used to establish respondents’ characteristics predicting the pandemic’s perceived impact on their lives. Descriptive and non-parametric statistics were performed using IBM SPSS 26 for Windows (IBM Corp, Armonk, NY).

## Results

### Characteristics of the respondents

Table 1 below shows the characteristics of the respondents. Two hundred and forty-three individuals attempted to complete the survey. Forty-five respondents were excluded because they completed less than 50% of the questionnaires. Consequently, one hundred and ninety-eight respondents were included in the analysis, 45.5% were male, and 54.5% were female. 69.3% of respondents were below 30 years of age, and 57.1% lived in Australia for fourteen years or less. In addition, 29.3% of the respondents identified themselves as permanent residents, 6.6% as temporary residents, and 64.1% as citizens. Many of the respondents, 55.6%, arrived in Australia on a Humanitarian Refugee visa. Most of the respondents were originally from Sub-Saharan Africa (36.9%), North Africa and the Middle East (27.8%) and Asia (26.8%). 57.1% of the respondents had university-level education. At the time of participation, respondents’ unemployment rate was 30.8%, and 61.1% had an annual income level of less than $50,000.

**Table 1:** Characteristics of the respondents (*N* = 198)

Variables	Frequency (%)
Gender	
Male	90 (45.5)
Female	108 (54.5)
Age	
18–20 years	73 (36.9)
21–29 years	64 (32.3)
30–39 years	37 (18.7)
40+ years	24 (12.1)
Region of origin	
Sub-Saharan Africa	72 (36.4)
North African & Middle East	55 (27.8)
Europe, America & Others	18 (9.1)
Asia	53 (26.8)
Visa on Arrival	
Humanitarian	110 (55.6)
Temporary	30 (15.2)
Others	28 (14.1)
Skilled	30 (15.2)
Residency status	
Permanent	58 (29.3)
Temporary	13 (6.6)
Citizen	127 (64.1)
Years lived in Australia	
0–5 years	55 (27.8)
6–14 years	58 (29.3)
15+ years	85 (42.9)
Number of children	
0–2 children	154 (77.8)
3–4 children	32 (16.2)
5+ children	12 (6.1)
Household size	
1–3 people	133 (67.2)
4–5 people	51 (25.8)
6+ people	14 (7.1)
Employment status	
Unemployed	61 (30.8)
Others	27 (13.6)
Employed	110 (55.6)
Work situation	
Temporary	49 (24.7)
Casual	62 (31.3)
Permanent	87 (43.9)
Work fraction	
0.2–0.4 FTE	42 (21.2)
0.5–0.7 FTE	66 (33.3)
0.8–1.0 FTE	90 (45.5)
Educational level	
Primary or Secondary	32 (16.2)
Some College/TAFE	53 (26.8)
University	113 (57.1)
Income level	
$0–$49,999	121 (61.1)
$50,000–$69,999	28 (14.1)
$70,000–$89,999	27 (13.6)
$90,000+	22 (11.1)

### Perceived experiences in selected livelihood items during COVID-19

**Fig. 1:**
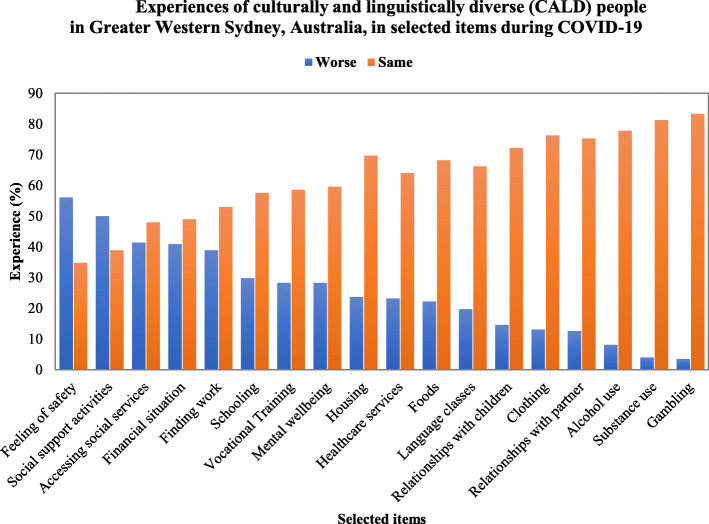
Experiences of culturally and linguistically diverse (CALD) people in Greater Western Sydney, Australia, in selected items during COVID-19

Figure 1 shows that safety was a top concern during the pandemic, with 56% of respondents rating their experience as “worse”. This was followed by accessing social support activities where 50% of respondents revealed their experience worsened during the pandemic. In addition, 41% of respondents felt their financial situation and experience accessing social services were “worse” during the pandemic. Finding work was rated “worse” by 39% of the respondents, and approximately one in four respondents felt their experience of attending schooling and vocational training were “worse” than before COVID-19. About one in four felt their mental wellbeing worsened. Some respondents revealed relationships, alcohol and substance use, and gambling issues during the pandemic, although many did not experience a considerable change in these items. 13% and 15% of the respondents revealed that relationships with their partners and children worsened during the pandemic, respectively. Additionally, a few respondents had engaged in alcohol and substance use and gambling, and they disclosed an increased use of these activities during the pandemic. 8% felt their alcohol use worsened, and 4% disclosed a rise in substance use and gambling.

### Perceived impacts of COVID-19 among respondents by experience in selected domains

The overall median score for the perceived impact of COVID-19 was 7, the mean was 6.5, and the variance was 6.2. There was a significant difference between respondents who rated their experience as “worse” than those who rated their experience as the “same” across most selected livelihood items. For example, Table 2 shows that the perceived impact of COVID-19 among respondents who rated their housing experience as “worse” (Mdn = 8) was higher than those who rated their experience as “same” (Mdn = 6). A Mann-Whitney U test showed that the observed difference was statistically significant, ∪(*N*_1_ = 58, *N*_2_ = 140) = 3136.5, *z* = 2.537, *p* = 0.011. Similar findings between the two groups were also observed for finding work, safety, clothing, financial situation, vocational training, accessing social services, relationship with the partner, and alcohol use. Interestingly, although most respondents rated social support group activities as the most impacted, this has no relationship to their overall perceived impact of the pandemic. Contrastingly, while a small proportion of participants rated their housing experience as ‘worse’, this shaped their overall perceived impact of the pandemic. Similarly, respondents who experienced fractured relationships with their partners reported significantly high impact of the pandemic, ∪(*N*_1_ = 43, *N*_2_ = 155) = 2652, *z* = 2.063, *p* = 0.039.

**Table 2:** Perceived impact of COVID-19 by experience in selected livelihood items

Selected livelihood items	T (N_**1**_, N_**2**_)	Mdn1	Mdn2	U	z-test	r	***p***-value
Housing situation	198 (58,140)	8	6	3136.5	2.537	0.180	0.011^a^
Finding work	198 (93,105)	7	6	3960	2.311	0.164	0.021^a^
Feeling of safety	198 (129,69)	7	6	3696.5	1.978	0.141	0.048^a^
Foods	198 (63,135)	8	6	3556	1.869	0.133	0.062
Clothing	198 (40,158)	8	6	2438	2.248	0.160	0.025^a^
Financial situation	198 (101,97)	7	6	3842.5	2.641	0.188	0.008^a^
Accessing social services	198 (103,95)	7	6	4075	2.046	0.145	0.041^a^
Social support activities	198 (121,77)	7	7	4388	0.694	0.049	0.488
Healthcare services	198 (67,131)	7	7	4231.5	0.415	0.029	0.678
Schooling	198 (82,116)	7	7	4414	0.868	0.062	0.385
Vocational Training	198 (78,120)	8	6	3695	2.52	0.179	0.012^a^
Language classes	198 (63,135)	7	7	3942	0.833	0.059	0.405
Relationships with partner	198 (43,155)	8	6	2652	2.063	0.147	0.039^a^
Relationships with children	198 (48,150)	7	7	3372	0.665	0.047	0.506
Mental wellbeing	198 (79,119)	7	6	4010	1.763	0.125	0.078
Alcohol use	198 (29,169)	8	6	1704.5	2.638	0.187	0.008^a^
Substance use	198 (18,180)	8	7	1352	1.165	0.083	0.244
Gambling	198 (12,186)	6	7	1055	0.32	0.023	0.749

*T* Total respondents, *N*_*1*_ Number of respondents rating their experience in the selected livelihood items as “worse”, *N*_*2*_ Number of respondents rating their experience in the selected livelihood items as “same”, *Mdn1* Median impact score for respondents who rated their experience in the selected livelihood items as “worse”, *Mdn2* Median impact score for respondents who rated their experience in the selected livelihood items as “same”, *U* Mann-Whitney U Statistics, *r* Effect Size

^a^Statistically significant difference at 0.05 significance level

### Predictors of perceived impact of COVID-19 among respondents

Respondents’ characteristics were examined to ascertain factors that could predict the perceived impact of the pandemic. Table 3 shows that respondents’ characteristics, including the region of origin, visa on arrival, residency status, number of children, employment status, work situation, and educational level, have significant relationships on the perceptions of the overall impact of COVID-19. For example, unemployed respondents were 3.53 (95% CI: 1.16–10.73, *p* = 0.026) times more likely to perceive a “high” impact of COVID-19 than those who were employed. Interestingly, among the employed respondents, casual employees reported less impact (OR = 0.2, 95% CI: 0.08–0.51, *p* = 0.001) than those who were permanent. Moreover, respondents who arrived in Australia on “Other” visa category showed a high perceived impact of the pandemic (OR = 2.68, 95% CI = 1.03–6.99, *p* = 0.044) than those who arrived on a skilled visa. Surprisingly, permanent but not temporary residents experienced a significantly high impact of perceived COVID-19 than those who were citizens (OR = 4.41, 95% CI: 1.69–11.53, *p* = 0.002). Another notable finding is that respondents with primary/secondary level education (OR = 0.19, 95% CI: 0.07–0.5, *p* = 0.001) and some college/TAFE (OR = 0.32, 95% CI: 0.14–0.69, *p* = 0.004) demonstrated less perceived impacts of COVID-19 than respondent with University level education.

**Table 3:** Exponentiated Ordinal Logistic Regression showing the perceived impact of COVID-19 among respondents (*n* = 198)

Variables	Categories	OR (95% CI)	Sig.
Gender	Female	.	.
	Male	1.3 (0.67,2.51)	0.438
Age	40+ years (ref)	.	.
	30–39 years	1.09 (0.36,3.31)	0.885
	21–29 years	1.46 (0.52,4.07)	0.473
	18–20 years	0.76 (0.27,2.16)	0.604
Regional origin	Asia (ref)	.	.
	Sub-Saharan Africa	0.92 (0.27,3.15)	0.894
	North Africa and Middle East	0.39 (0.17,0.94)	0.036^a^
	Europe, Americas & Others	0.3 (0.13,0.72)	0.007^a^
Visa on Arrival	Skilled (ref)	.	.
	Humanitarian	1.43 (0.47,4.41)	0.529
	Temporary	1.32 (0.42,4.18)	0.641
	Others	2.68 (1.03,6.99)	0.044^a^
Residency status	Citizen (ref)	.	.
	Temporary	4.43 (0.86,22.99)	0.076
	Permanent	4.41 (1.69,11.53)	0.002^a^
Years in Australia	15+ years (ref)	.	.
	6–14 years	1.61 (0.75,3.43)	0.222
	0–5 years	0.4 (0.13,1.22)	0.108
Number of children	5+ children (ref)	.	.
	3–4 children	2.36 (0.56,9.98)	0.245
	0–2 children	4.21 (1.1,16.2)	0.036^a^
Household size	6+ people (ref)	.	.
	4–5 people	0.65 (0.18,2.41)	0.518
	1–3 people	0.31 (0.09,1.11)	0.072
Employment status	Employed (ref)	.	.
	Unemployed	3.53 (1.16,10.73)	0.026^a^
	Others	2.34 (0.9,6.07)	0.081
Work situation	Permanent (ref)	.	.
	Temporary	0.5 (0.2,1.28)	0.148
	Casual	0.2 (0.08,0.51)	0.001^a^
Work Fraction	0.8–1.0 FTE (ref)	.	.
	0.5–0.7 FTE	1.1 (0.48,2.55)	0.824
	0.2–0.4 FTE	1.29 (0.42,3.98)	0.654
Education level	University (ref)	.	.
	Some College/TAFE	0.32 (0.14,0.69)	0.004^a^
	Primary/Secondary	0.19 (0.07,0.5)	0.001^a^
Income	$90,000+ (ref)	.	.
	$60,000–$89,999	1.66 (0.51,5.39)	0.401
	$50,000–$69,999	2 (0.6,6.66)	0.261
	$0–$49,999	1.31 (0.41,4.23)	0.653

Dependent variable – Impact of COVID-19, *Ref* Reference category, *OR* Odd ratio

^a^Statistically significant difference at 0.05 significance level

## Discussion

The current study explored the impacts of COVID-19 among people from culturally and linguistically diverse (CALD) backgrounds in Western Sydney, New South Wales, Australia. It found that although 57.1% of the respondents had university-level education, 20.8% were unemployed, and 41.1% had an annual income of less than $50,000. This unemployment rate was much higher than the 7.2% and 6.9% rates in the New South Wales and Australia population, respectively, at the time of the data collection in September 2020 [36]. Additionally, the finding showed that COVID-19 had notable impacts on people’s livelihood. Respondents’ feeling of safety was disrupted the most by the pandemic. This disruption is followed by social support group activities, accessing social services, financial situation, finding work, attending schools, mental wellbeing, and housing. The finding highlights the disruptions caused by COVID-19 on important services, yet services of these kinds are vital for CALD communities in Australia [37]. The experience of respondents in this study corroborates the experiences of migrants in other countries. For example, several studies suggest that migrants experienced economic inequality the most and significant poverty during the COVID-19 pandemic [38–41]. The disruptions that emerged in the present study are concerning and might exacerbate the social disadvantages experienced by many CALD people in Australia [42, 43].

This study found that changes in some life domains were associated with a significantly greater overall impact of the pandemic on participants. For example, though only a relatively small proportion of participants (20%) reported that their housing situation had worsened during the pandemic, this was associated with an overall significant and substantially greater impact of the pandemic on the person’s life. A similar observation was found for employment and relationships with partners. By contrast, though many participants (50%) reported experiencing worse social support activities, this was not associated with greater impact of the pandemic on their livelihood. Current literature on the impacts of COVID-19 aligning with the finding in this study found an increased mental and psychological distress because of challenges (such as unemployment) caused by the pandemic [44]. This suggests that housing, employment and relationships experiences are important social determinants of health, shaping individual self-outlook in this case.

The reported finding is congruent with existing literature that theorized the harmful impacts of COVID-19 on couples’ relationships [45]. This finding aligns with an earlier study that found increased intimate partner violence during COVID-19 because of lockdown and compromised access to specialized services [46]. Thus, COVID-19 intensified partner relationships issues in this community, which was already a concern and needed addressing before the pandemic [47]. Moreover, housing problems among CALD people during the pandemic has been well acknowledged [48, 49]. Furthermore, respondents’ identified disruption in employment was not surprising because many businesses were closed during the pandemic, and many people lost their jobs and livelihood [50]. Other literature has also reported these issues in other countries [51, 52]. The employment disruptions found in this study and the evidence from previous literature underline the daily struggle to make ends meet experienced by many individuals. While COVID-19 has undoubtedly contributed to the current employment and housing situations, it has also acted as a catalyst to expose these rooted disadvantages in the CALD community in Australia and elsewhere [18, 20, 42, 53].

Additionally, this study found that some respondents’ characteristics were significant predictors of the overall perceived impact of COVID-19. For instance, unemployed respondents were more likely to perceive a “high” impact of COVID-19 than employed. Interestingly, among employed respondents, casual employees reported less impact than permanent employees. This observation suggests that casual employees still had their job or those permanently employed were distressed by the real possibility that they might lose their jobs and experience financial shocks [54]. Finally, the study found a mixed result for residency status, with permanent but not temporary residents experiencing the most significant impact of COVID-19 than those who were citizens. Many permanent residents in this study arrived in Australia through a humanitarian program. They possibly did not have the financial resources needed during the pandemic compared to temporary residents who arrived mainly as international students and holidaymakers and could have had the financial support base.

Another notable finding is that respondents with primary/secondary level education and some college/TAFE experienced significantly lower impacts of COVID-19 than respondents with University level education. This finding could be attributed to health literacy relating to the pandemic. On the one hand, respondents with university-level education might have had better health literacy knowledge to discern information about the pandemic, follow the regular update on preventative measures contributing to their perceived impacts than those with low education levels [55]. On the other hand, although health literacy is a social determinant of health and is associated with high education, income, employment and high socio-economic status, evidence suggests that even people with higher education can experience low health literature in the event of a new health condition, like COVID-19 [56].

The current study has some limitations. The study sample size was relatively small, and the findings need to be interpreted with caution. Also, we did not include members of the non-CALD community in the study. Therefore, the data does not represent the whole of NSW, and it is systematically different because of the “high” population of people born overseas living within the Greater Western Sydney. Accordingly, the findings cannot be directly compared with the larger NSW or Australian population. Additionally, most respondents had university-level education and could read and write English, so the findings need to be viewed with this understanding. They may not be generalizable for people who do not speak English well because their experience of COVID-19 may have a different context. For this reason, the identified experience of disruptions and the perceived impact of the pandemic reported in this study could have been underreported. Also, this study is unlikely to be representative of the CALD population because of our convenience sampling approach [31].

### Implications for policymakers and service providers

Despite Australia’s success in supporting the resettlement of people from non-English speaking backgrounds and non-western cultures and providing for their specific needs, the unusual circumstances that emerged due to COVID-19 highlight gaps in the system and suggested the domains where current and future needs may be building. A coordinated public and private sector response might help address these gaps and provide greater opportunities for members of the CALD communities to build on existing strengths and develop a greater capacity for self-reliance and economic, social and health development, and prevent the risk of ill health later in lives arising from the disruptions caused by COVID-19 [57]. The resilience observed in the study population, which may account for the low number of individuals reporting negative responses to the situation, like increased drinking, substance use or gambling, is a strength that shows adaptability in the CALD communities of Western Sydney. This is an important personal and community resource that should not be wasted. Nonetheless, some domains cannot be addressed by individuals or communities alone where change can only be effected at the level of government. This study points out the importance of some of these system issues to the CALD population based on the affected communities’ experiences and perceptions. It also emphasizes the fact that all communities have strengths, even if they also experience disparities. Effective policy and initiatives for the future will leverage these strengths while addressing the weak points in the system.

## Conclusion

The study respondents reported disruptions in employment, housing and relationships with their partners. These disruptions are associated with significant impacts of the pandemic, which is not surprising because members of these communities are known to experience economic, social, and health disparities compared to the Australian population as a whole. The finding suggests a need for enhanced policy and initiatives designed to meet the needs of the CALD population, particularly concerning the areas reported to have been disrupted the most by changes associated with COVID-19. The COVID-19 situation, then, can serve as a lens to focus attention on the most disrupted domains as experienced by Western Sydney’s CALD communities. Further longitudinal research is required to identify the long term impacts of the disruptions reported in this study. This will allow resources to be more effectively directed toward unmet needs while supporting resilience and agency within and among CALD communities in Sydney and elsewhere.

## Supplementary Information

**Additional file 1.** Relevant items developed for the current study.

## Data Availability

The data that support the findings of this study are available from SydWest Multicultural Services, but restrictions apply to the availability of these data, which were used under license for the current study, and so are not publicly available. However, data are available from the authors (WM, CM) upon reasonable request and with permission of SydWest Multicultural Services.
