# Supplementary material for: A cross-sectional study of COVID-19 impacts in culturally and linguistically diverse communities in greater Western Sydney, Australia
Source: BMC Public Health. 2021 Nov 13;21:2081. doi: 10.1186/s12889-021-12172-y (PMC8590135; doi:10.1186/s12889-021-12172-y)
Supplement: Supplementary file 1 — Additional file 1. Relevant items developed for the current study. [file 12889_2021_12172_MOESM1_ESM.docx]

**Appendix A: Survey Instrument - Impacts of COVID-19 in migrant and refugee communities in the Greater Western Sydney Region, NSW, Australia**

1 What is your age in years?

▼ 18 (1) ... 80 (63)

2 What is your gender? (Choose one answer)

- 1 Male (1)
- 2 Female (2)
- 3 Other (3)

3 How many years have you lived in Australia?

▼ 1 (1) ... 50 (50)

4 What is your race/ethnicity?

- White (1)
- Black (2)
- Oriental (Asian) (3)
- Hispanic (4)
- Oceania (5)
- Other (6)

|  |
| --- |

5 What is your country of birth?

________________________________________________________________

6 What is your residence status in Australia? (Choose one of the following answers)

- Australian citizen (1)
- Permanent residents (2)
- Temporary visa (3)
- I don’t know (4)
- Other (5)

7 How many people live with you in the same house, including you?

▼ 1 (1) ... 20 (20)

8 How many children (0-17 years) live with you?

▼ 1 (1) ... 15 (15)

9 What is your highest educational level? Choose one answer

- No education (1)
- Primary school (2)
- Secondary school (3)
- Some college/TAFE (4)
- University education (5)
- Other (6)

10 My current work situation is (Choose one of the following answers)

- Work normally (1)
- Sent home to work remotely (2)
- Sent home and receiving unemployment allowance (3)
- Sent home without pay and not receiving unemployment allowance (4)
- I was already unemployed before COVID-19 (5)
- Others (6)

11 If you are employed, what is your income range per year?

- $30,000 or less (1)
- $40,000-$49,999 (2)
- $50,000-$59,999 (3)
- $60,000-$69,999 (4)
- $70,000-$79,999 (5)
- $80,000-$89,999 (6)
- $90,000-$99,999 (7)
- $100,000+ (8)

12 At what fraction are you currently employed? If you are full-time, select 1.0 FTE (full-time equivalent)

- 1.0 FTE (FULL TIME 5 days a week) (1)
- 0.9 FTE (4.5 days a week) (2)
- 0.8 FTE (4 days a week) (3)
- 0.7 FTE (3.5 days a week) (4)
- 0.6 FTE (3 days a week) (5)
- 0.5 FTE (2.5 days a week) (6)
- 0.4 FTE (2 days a week) (7)
- 0.3 FTE (1.5 days a week) (8)
- 0.2 FTE (1 day a week) (9)

13 How do you describe your employment status?

- Full-time temporary (1)
- Full-time permanent (2)
- Full-time casual (3)
- Part-time casual (4)
- Part-time permanent (5)

14 On a scale of 1 to 10, how much do the COVID-19 measures impact your lives? 1 = not at all 10 = extremely

|  | 0 | 1 | 2 | 3 | 4 | 5 | 6 | 7 | 8 | 9 | 10 |
| --- | --- | --- | --- | --- | --- | --- | --- | --- | --- | --- | --- |

| Impact on your lives () | 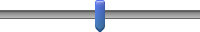 |
| --- | --- |

15 On a scale from 1 to 10, how socially isolated do you feel now? 1 = not at all, 10 = extremely

|  | 0 | 1 | 2 | 3 | 4 | 5 | 6 | 7 | 8 | 9 | 10 |
| --- | --- | --- | --- | --- | --- | --- | --- | --- | --- | --- | --- |

| Feel socially isolated | 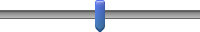 |
| --- | --- |

16 On a scale of 1 to 10, how worried are you about the COVID-19 crisis? 1= not at all, 10= extremely

|  | 0 | 1 | 2 | 3 | 4 | 5 | 6 | 7 | 8 | 9 | 10 |
| --- | --- | --- | --- | --- | --- | --- | --- | --- | --- | --- | --- |

| Feel worried | 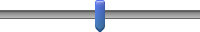 |
| --- | --- |

17 Since the COVID-19 crisis, how do you rate your experience in the following lives domains?

|  | Worse than before (1) | Same as before (2) | Not applicable (4) |
| --- | --- | --- | --- |
| Housing situation (1) |  |  |  |
| Accessing work (2) |  |  |  |
| Feelings of safety (3) |  |  |  |
| Food (4) |  |  |  |
| Clothes (5) |  |  |  |
| Financial means (6) |  |  |  |
| Accessing social services (7) |  |  |  |
| Social support group activities (8) |  |  |  |
| Medical care (9) |  |  |  |
| Schooling (10) |  |  |  |
| Vocational training (11) |  |  |  |
| English language classes (12) |  |  |  |
| Relationship with my partner/husband/wife (13) |  |  |  |
| Relationship with my children (14) |  |  |  |
| Issues with mental health (15) |  |  |  |
| Alcohol consumptions (16) |  |  |  |
| Substance use (17) |  |  |  |
| Gambling (18) |  |  |  |
